# Broad ranges of investment configurations for renewable power systems, robust to cost uncertainty and near-optimality

**DOI:** 10.1016/j.isci.2023.106702

**Published:** 2023-04-21

**Authors:** Fabian Neumann, Tom Brown

**Affiliations:** 1 Department of Digital Transformation in Energy Systems, Institute of Energy Technology, Technische Universität Berlin (TUB), Einsteinufer 25 (TA 8), 10587 Berlin, Germany; 2 Institute for Automation and Applied Informatics (IAI), Karlsruhe Institute of Technology (KIT), Hermann-von-Helmholtz-Platz 1, 76344 Eggenstein-Leopoldshafen, Germany

**Keywords:** Energy resources, Energy policy, Energy management, Energy Modelling, Energy Systems

## Abstract

Achieving ambitious ${CO}_{2}$ emission reduction targets requires energy system planning to accommodate societal preferences, such as transmission reinforcements or onshore wind parks, and acknowledge uncertainties in technology cost projections among many other uncertainties. Current models often solely minimize costs using a single set of cost projections. Here, we apply multi-objective optimization techniques in a fully renewable European electricity system to explore trade-offs between system costs and technology deployment for electricity generation, storage, and transport. We identify ranges of cost-efficient capacity expansion plans incorporating future technology cost uncertainties. For example, we find that some grid reinforcement, long-term storage, and large wind capacities are important to keep costs within 8% of least-cost solutions. Near the cost optimum a technologically diverse spectrum of options exist, allowing policymakers to make trade-offs regarding unpopular infrastructure. Our analysis comprises 50,000+ optimization runs, managed efficiently through multi-fidelity surrogate modeling techniques using sparse polynomial chaos expansions and low-discrepancy sampling.

## Highlights

• Uses open electricity system model PyPSA-Eur to explore near-optimal trade-offs
• Wide range of options to design renewable power systems within 8% of optimum
• Many of these options are robust to uncertain technology cost developments
• Degrees of freedom can help policymakers to circumvent public acceptance issues

## Graphical abstract

**Figure:**
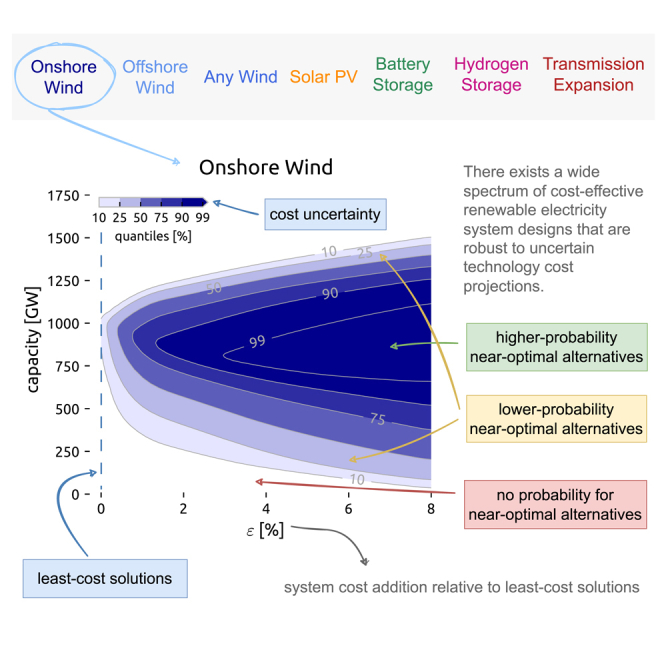

## Introduction

Energy system models have become a pivotal instrument for policy-making to find cost-efficient system layouts that satisfy ambitious climate change mitigation targets. But even though they have proliferated in spatial, temporal, technological, and sectoral detail and scope in recent years, least-cost optimization models can easily give a false sense of exactness.^1^^,^^2^ Frequently, they present just a single least-cost solution for a single set of cost assumptions, which not only neglects uncertainties inherent to technology cost projections, which can have a strong effect on the results of capacity expansion models,^3^^,^^4^^,^^5^ but also hides a wide array of alternative solutions that are equally feasible and only marginally more expensive.^6^^,^^7^^,^^8^

Trade-offs revealed by deviating from least-cost solutions are extremely attractive for policymakers because they allow them to make decisions based on non-economic criteria without affecting the cost-effectiveness of the system. Knowing that many similarly costly but technologically diverse solutions exist helps to accommodate political and social dimensions that are otherwise hard to quantify; for instance, rising public opposition toward new transmission lines and onshore wind turbines or an uneven distribution of new infrastructure.^8^^,^^9^^,^^10^

Techniques like multi-objective optimization and modeling-to-generate-alternatives (MGAs) are designed to find such near-optimal solutions. They have been applied to investment planning models of the European,^6^ the Italian,^7^ and the United States power system,^11^ pathways to decarbonize the power system of the United Kingdom,^12^ the European sector-coupled energy system,^13^ a single-node energy model of Germany,^14^ and global integrated assessment models^15^ and were combined with a quick hull algorithm to span a polytope of low-cost solutions for a single set of cost parameters.^16^

However, most of the studies above only use a central cost projection for each considered technology. Recent decades have shown that many of these projections contain a high level of uncertainty, particularly for fast-moving technologies like solar, wind, batteries, and hydrogen storage.^17^^,^^18^^,^^19^ This uncertainty propagates through the model to strongly affect the optimal and near-optimal system compositions, thus undermining any analysis of the trade-offs. Hence, it is crucial that apparent compromises are rigorously tested for robustness to technology cost uncertainty to raise confidence in conclusions about viable, cost-effective power system designs. To thoroughly sweep this uncertainty space, we can avail of previous studies on multi-dimensional global sensitivity analysis techniques in the context of least-cost optimization.^3^^,^^20^^,^^21^^,^^22^^,^^23^ In this context, it is also important to note that this methodology can also be used to address many further uncertainties, for instance, regarding weather variability between years, demand projections, and the level of cross-sectoral integration that can be realized.

In this paper, we bring together near-optimal analysis with global sensitivity sweeps over uncertainty. This allows us to systematically explore more robust trade-offs near the cost optimum of a fully renewable European electricity system model, PyPSA-Eur,^24^ and investigate how they are affected by uncertain technology cost projections. We evaluate compromise solutions between system cost and technology choices by minimizing and maximizing the use of on- and offshore wind, solar photovoltaics, transmission, batteries, and hydrogen storage in order to identify near-optimal alternatives that are no more than 8% more expensive than the least-cost option. This discretionary choice is low enough to argue that such solutions are nearly cost optimal and high enough such that most trade-offs have flattened out at this point. However, more expensive solutions may still be acceptable to the public, in particular when they are more widely accepted or if they could be implemented more quickly. We also show examples of trade-offs for pairs of technologies and a chosen allowed cost penalty of 6%; namely between wind and solar, battery and hydrogen storage, and offshore and onshore wind. For this research, we solve numerous spatially and temporally explicit long-term investment planning problems that coordinate generation, transmission, and storage investments subject to multi-period linear optimal power flow constraints. The capacity optimization is supplemented with global sensitivity analysis methods to account for a wide range of cost projections for wind, solar, battery, and hydrogen storage technologies. These cost projections are assumed to be uniformly distributed based on ranges from the Danish Energy Agency (DEA) for the year 2050.^25^

**Figure 1:**
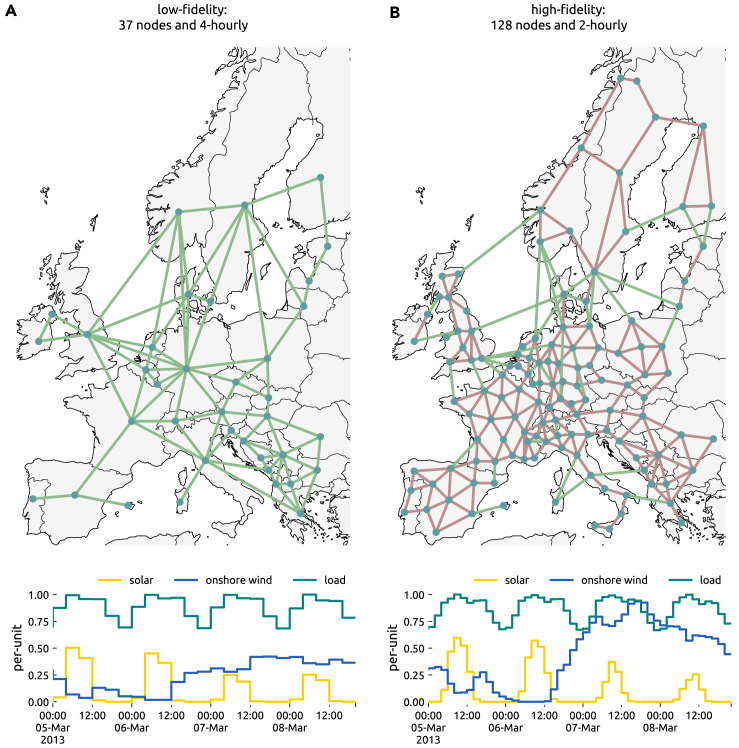
Spatial and temporal resolution of the low- and high-fidelity model (A and B) (A) Low-fidelity model with 37 nodes and 4-hourly resolution, (B) high-fidelity model with 128 nodes and 2-hourly resolution. Green lines represent controllable HVDC lines. Red lines represent HVAC lines. Examples for capacity factors for wind and solar are shown for four days in March at the northernmost node in Germany, alongside the normalized load profile.

**Figure 2:**
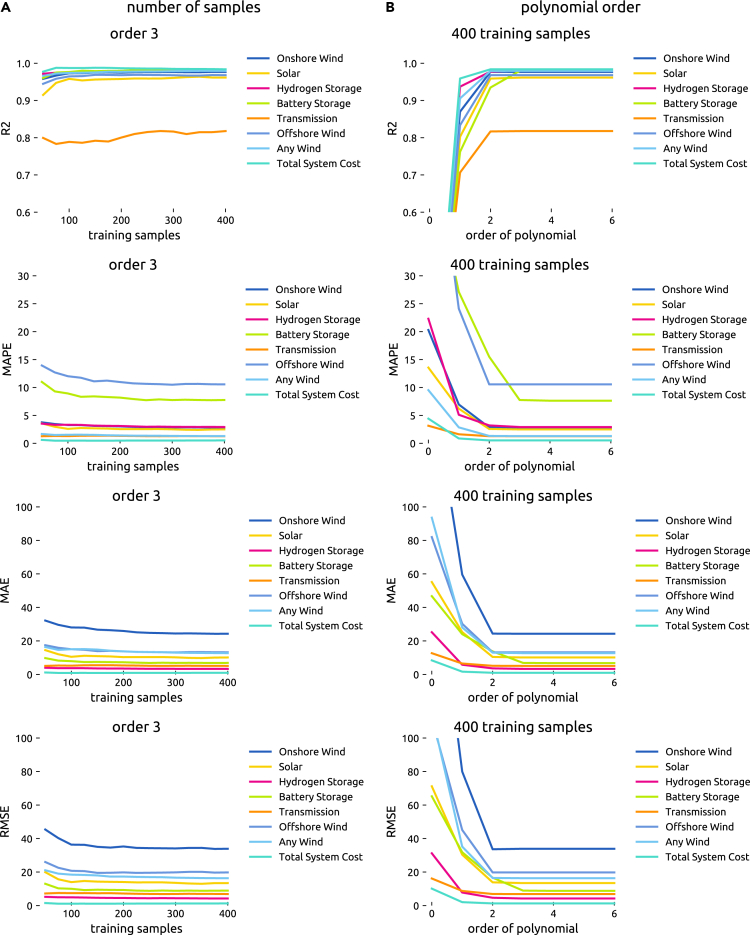
Cross-validation errors (A) Cross-validation errors by output for varying sample sizes. (B) Cross-validation errors by output for varying polynomial orders of least-cost low-fidelity surrogate models.

To handle the high computational burden incurred by searching for near-optimal alternatives alongside evaluating many different cost parameter sets, we employ multi-fidelity surrogate modeling techniques, based on sparse polynomial chaos expansion, that allow us to merge results from one simpler and another more detailed model. One covers 37 regions at 4-hourly and the other 128 regions at 2-hourly resolution over a full year (see Figure 1 and multi-fidelity approach in surrogate modeling for details). This approach has been proven very effective in Tröndle et al.^3^ Heavy parallelization with high-performance computing infrastructure allows us to solve more than 50,000 resource-intensive optimization problems which, in combination with surrogate modeling, can span a probabilistic space of near-optimal solutions with respect to cost uncertainties rather than putting single technology cost futures into the foreground (see also Figure 2). While the methodology is general enough to be applied to other uncertainties like interannual weather variability or demand projections, our application addresses the probabilistic space of technology cost projections.

In this way, we are able to present alternative solutions beyond least cost that have a high chance of involving a limited cost increase, just as we identify regions that are unlikely to be cost efficient for realizing a fully renewable electricity system. We derive both ranges of options and technology-specific capacity expansion ranges that are not affected by cost uncertainty and should be met to keep the total system cost within a specified range, given the model setup. Our results show that indeed many such similarly costly but technologically diverse solutions exist regardless of how technology cost developments will unfold within the considered ranges.

We approach the presentation of these results in two steps: we first show the propagation of uncertainties in least-cost solutions, including a global sensitivity analysis that identifies the most influential cost parameters. We then gradually expand the uncertainty analysis to the space of nearly cost-optimal solutions.

## Results

### System cost and capacity distribution of least-cost solutions

**Figure 3:**
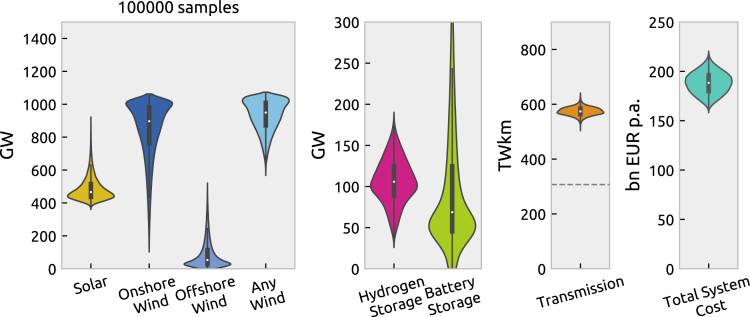
Distribution of system cost, generation, storage, and transmission in least-cost solutions The dashed line in the transmission line chart indicates today’s existing transmission capacities for comparison. Violin range is limited to the range of observed data. Boxplots show median, interquartile range, and upper/lower quartile ±1.5 times the interquartile range.

We explore the impacts of cost uncertainty in a spatially and temporally resolved model of the European power system with fully renewable generation and zero direct carbon dioxide emissions. Based on sampling the uncertainty of cost inputs given by the DEA (Table 1),^25^ the total annual power system costs vary between 160 and 220 bn€/a, as displayed in Figure 3. This means the most pessimistic cost projections entail about 40% higher cost than the most optimistic projections. All least-cost solutions build more than 350 GW solar and 600 GW wind, but none more than 1100 GW of wind or more than 950 GW of solar. While wind capacities tend to cluster toward higher values, solar capacities tend toward lower values. We observe that least-cost solutions prefer onshore over offshore wind, yet onshore wind features the highest uncertainty range alongside battery storage. The cost optimum gravitates toward hydrogen storage rather than battery storage unless battery storage becomes very cheap. In the uncertainty space sampled, there are no least-cost solutions without the long-duration storage provided by hydrogen, only some without battery storage. Transmission network expansion is least affected by cost uncertainty and consistently doubled compared to today’s capacities to achieve a fully renewable electricity system.

**Table 1:** Technology cost uncertainty using optimistic and pessimistic assumptions from the Danish Energy Agency

Technology	Lower CAPEX	Upper CAPEX	Unit	Source
Onshore Wind	800	1190	EUR/kW	DEA^25^
Offshore Wind	1420	1950	EUR/kW	DEA^25^
Solar	420	620	EUR/kW	DEA^25^
Battery	316	1306	EUR/kW	DEA^25^
Hydrogen	668	2002	EUR/kW	DEA^25^

Although these results outline the extent to which cost uncertainty affects cost-optimal designs, the insights from the observed ranges are limited because there is considerable flexibility beyond the least-cost solutions and acknowledge structural modeling uncertainties, such as social constraints to the expansion of grids and wind turbines. Moreover, the pure distribution of outputs does not yet convey information about how sensitive results are to particular cost assumptions. But knowing the technologies for which lowering overnight costs has a significant impact is important to promote technological learning in that direction.

### Local parameter sweeps and global sensitivity indices

**Figure 4:**
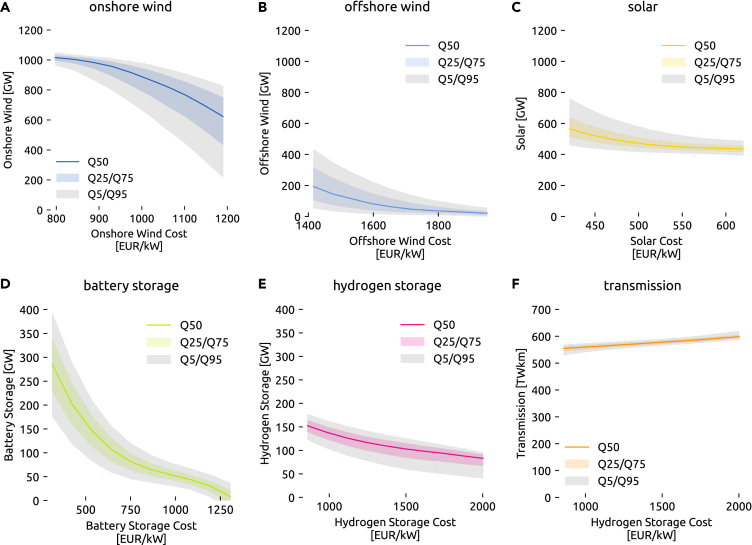
Sensitivity of capacities toward their own technology cost (A–F) (A) Onshore wind, (B) offshore wind, (C) solar, (D) battery storage, (E) hydrogen storage, (F) transmission. The median (Q50) alongside the 5%, 25%, 75%, and 95% quantiles (Q5–Q95) display the sensitivity subject to the uncertainty induced by other cost parameters. Dots represent samples of the high-fidelity model runs.

Therefore, Figure 4 expands the previous view by additionally showing how the cost of a technology influences its deployment while displaying the remaining uncertainty induced by other cost parameters. The overall tendency is easily explained: the cheaper a technology becomes, the more it is built. However, changes of slope and effects on the uncertainty range as one cost parameter is swept are insightful still. For instance, Figure 4 reveals that battery storage becomes significantly more attractive economically once its cost falls below 750 EUR/kW (including 6-h energy capacity at full power output), while hydrogen storage (including electrolysis, fuel cell, and underground storage with an energy-to-power ratio of 168 h) features a steady slope. A low cost of onshore wind makes building much onshore wind capacity attractive with low uncertainty, whereas if onshore wind costs are high how much is built greatly depends on other cost parameters. The opposite behavior is observed for offshore wind and solar. The cost of hydrogen storage mostly causes the limited uncertainty about cost-optimal levels of grid expansion. As the cost of hydrogen storage falls, less grid reinforcement is chosen.

But since the presented sensitivity of capacities toward their own cost only exhibits a fraction of all sensitivities, we further apply a more systematic variance-based global sensitivity analysis, which has been applied in energy systems analysis, e.g., in Tröndle et al.^3^ and Mavromatidis et al.^20^ Sensitivity indices, or Sobol indices, attribute the observed output variance to each input.^26^ For our application, the Sobol indices can, for instance, tell us which technology cost contributes the most to total system cost or how much of a specific technology will be built. The first-order Sobol indices describe the share of output variance due to variations in one input alone averaged over variations in the other inputs. Total Sobol indices also consider higher-order interactions, which are greater than 100% if the relations are not purely additive or independent.

**Figure 5:**
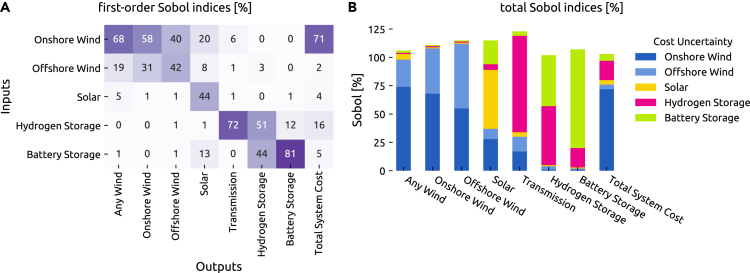
First-order and total Sobol indices (A and B) (A) First-order Sobol indices, (B) total Sobol indices. These sensitivity indices attribute output variance to random input variables and reveal which inputs the outputs are most sensitive to. The first-order Sobol indices quantify the share of output variance due to variations in one input parameter alone. The total Sobol indices further include interactions with other input variables. Total Sobol indices can be greater than 100% if the contributions are not purely additive.

The first-order and total Sobol indices for least-cost solutions in Figure 5 show that the total system cost is largely determined by how expensive it is to build onshore wind capacity, followed by the cost of hydrogen storage. The amount of wind in the system is almost exclusively governed by the cost of onshore and offshore wind parks. Other carriers yield a more varied picture. The cost-optimal solar capacities additionally depend on onshore wind and battery costs. The amount of hydrogen storage is influenced by battery and hydrogen storage cost alike. Although there are noticeable higher-order effects, which are most extensive for transmission, the first-order effects dominate. Strikingly, the volume of transmission network expansion strongly depends on the cost of hydrogen storage. This can be explained because they both compete to balance out the large weather systems crossing the continent, which cause in particular wind variations. Hydrogen storage can balance the multi-week transit of weather systems in time, whereas transmission networks can smooth them in space. While hydrogen storage typically balances multi-week variations in time, continent-spanning transmission networks exploit the circumstance that, as weather systems traverse the continent, it is likely always to be windy somewhere in Europe.

### Fuzzy near-optimal corridors with increasing cost slack

So far, we quantified the output uncertainty and analyzed the sensitivity toward inputs at least-cost solutions only. However, it has been previously shown that even for a single cost parameter set a wide array of technologically diverse but similarly costly solutions exists.^6^^,^^7^^,^^13^^,^^16^ We now examine how technology cost uncertainty affects the shape of the space of near-optimal alternatives within 8% of the least-cost solution. We do not extend beyond this value because the rate of change of most Pareto fronts has considerably reduced at that point, while we acknowledge that higher cost penalties may still be acceptable.

**Figure 6:**
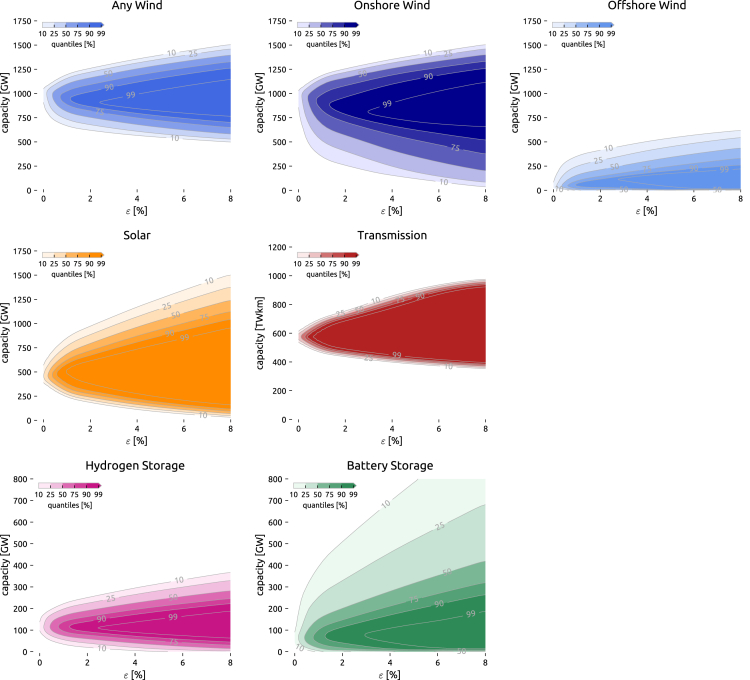
Space of near-optimal solutions by technology under cost uncertainty For each technology and cost sample, the minimum and maximum capacities obtained for increasing cost penalties ε form a cone of an upper and a lower Pareto front, starting from a common least-cost solution. By arguments of convexity, the capacity ranges contained by the cone can be near optimal and feasible, given a degree of freedom in the other technologies. From optimization theory, we know that the cones widen up for increased slacks. As we consider technology cost uncertainty, the cone will look slightly different for each sample. The contour lines represent the frequency a solution is inside the near-optimal cone over the whole parameter space. This is calculated from the overlap of many cones, each representing a set of cost assumptions. Due to discrete sampling points in the ε-dimension, the plots further apply quadratic interpolation and a Gaussian filter for smoothing.

By identifying feasible alternatives common to all, few or no cost samples, in Figure 6 we outline low-cost solutions common to most parameter sets (e.g., above 90% contour) as well as system layouts that do not meet low-cost criteria for nearly no technology cost samples for varying ϵ. For each technology and cost sample, the minimum and maximum capacities obtained for increasing cost penalties ϵ form a cone of an upper and a lower Pareto front, starting from a common least-cost solution. These Pareto fronts delineate boundaries beyond which neither reducing system cost nor extremizing the capacity of a technology can be improved without depressing the other. By arguments of convexity, the capacity ranges contained by the cone can be near optimal and feasible, given a degree of freedom in the other technologies. From optimization theory, we also know that the cones widen up for increased slacks. As we consider technology cost uncertainty, the cone will look slightly different for each sample causing the fuzziness of the boundaries. The contour lines represent the frequency with which a solution is inside the near-optimal cone over the whole parameter space. This is calculated from the overlap of many cones, each representing a different set of cost assumptions. The wider the displayed contour lines are apart, the more uncertainty exists about the borders. The closer contour lines are together, the more specific the limits are despite the cost uncertainty. The height of the quantiles quantifies flexibility for a given level of certainty and slack; the angle presents information about the sensitivity toward cost slack.

From the fuzzy upper and lower Pareto fronts in Figure 6, it can be seen that for our scenarios, building 900 GW of wind capacity is highly likely possible within 3% of the optimum and that conversely building less than 600 GW has a low chance of being near the cost optimum with our model setup. Only a few solutions can forego onshore wind entirely and remain within 8% of the cost optimum, whereas it appears to be likely possible to build a system without offshore wind at a cost penalty of 4% at most. On the other hand, more offshore wind generation seems equally possible. Unlike for onshore wind, where it is more uncertain how little can be built, uncertainty regarding offshore wind deployment exists about how much can be built so that costs remain within a pre-specified range. For solar, the range of options within 8% of the cost optimum at 90% certainty is very wide. Anything between 100 GW and 1000 GW appears feasible as long as other substituting technologies are built and suitably sited. In comparison to onshore wind, the uncertainty about minimal solar requirements is smaller.

The level of required transmission expansion is least affected by the cost uncertainty. To remain within the pre-defined ϵ=8%, it is just as likely feasible to plan for moderate grid reinforcement by 30% as is initiating extensive remodeling of the grid by tripling the transmission volume compared to what is currently in operation. One reason for this is perhaps that cost uncertainty on building new transmission lines was not included as it is a quite mature technology. These results indicate that in any of the cases considered some transmission reinforcement to balance renewable variations across the continent appears to be essential. Hydrogen storage, symbolizing medium- to long-term storage, also is a vital technology in many cases. In a model with increased cross-sectoral integration, this role could also likely be taken over by thermal storage or other power-to-X conversion processes. Some short- to medium-term balancing needs might also be covered by demand-side management. At ϵ=8%, only 25% of cost samples require no long-term storage; namely when battery costs are exceptionally low. Overall, 90% of cases appear to function without any short-term battery storage while the system cost rises by 4% at most. However, especially battery storage exhibits a large degree of freedom to build more given the high cost uncertainty reported in the DEA technology database.^25^

### Probabilistic near-optimal feasible space in two technology dimensions

**Figure 7:**
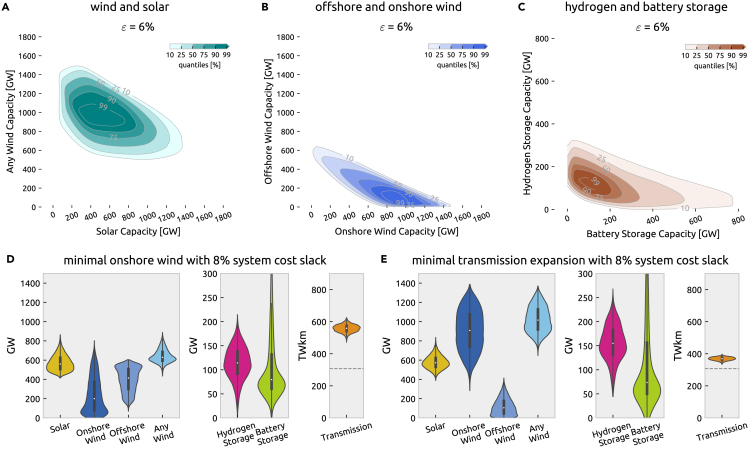
Space of near-optimal solutions by selected pairs of technologies under cost uncertainty (A–C) Just like in Figure 6, the contour lines depict the overlap of the space of near-optimal alternatives across the parameter space. It can be thought of as the cross-section of the probabilistic near-optimal feasible space for a given ϵ in two technology dimensions and highlights that the extremes of two technologies from Figure 6 cannot be achieved simultaneously. (D and E) Plots show the distribution of total system cost, generation, storage, and transmission capacities for two near-optimal search directions with ϵ=8% system cost slack. The dashed line in the transmission line chart indicates today’s existing transmission capacities.

The fuzzy cones from Figure 6 look at trade-offs between system cost and single techologies, assuming that the siting and deployment of other technologies can be heavily optimized. But as there are dependencies between the technologies, in Figure 7 we furthermore evaluate trade-offs between technologies for three selected pairs of technologies at an example fixed system cost increase of ϵ=6% for illustration, addressing which *combinations* of wind and solar capacity, offshore and onshore turbines, and hydrogen and battery storage are likely to be cost efficient.

First, Figure 7A addresses constraints between wind and solar. The upper right boundary exists because building much of both wind and solar would be too expensive for the given budget. The absence of solutions in the bottom left corner means that building too little of any wind or solar does not suffice to generate enough electricity. From the shape and contours, we see a high chance that building 1000 GW of wind *and* 400 GW of solar is within 6% of the cost optimum for the scenarios at hand. On the other hand, building less than 200 GW of solar and 600 GW of wind is unlikely to yield a low-cost solution in our model setup. In general, minimizing the capacity of both primal energy sources will shift capacity installations to high-yield locations even if additional network expansion is necessary and boost the preference for highly efficient storage technologies. Overall, we can conclude from this that, even considering combinations of wind and solar, a wide space of low-cost options exists with moderate to high likelihood, although the range of alternatives is shown to be more constrained.

The trade-off between onshore wind and offshore wind is illustrated in Figure 7B. Here, the most certain area is characterized by building more than 600 GW onshore wind and less than 250 GW offshore wind capacity for our electricity-only scenarios. However, there are some solutions with high substitutability between onshore and offshore wind, shown in the upper left bulge of the contour plot. Compared to wind and solar, the range of near-optimal solutions is even more constrained. The key role of energy storage in a fully renewable system is underlined in Figure 7C. Around 50 GW of power capacity of each is at least needed in any of the considered cases, while highest likelihoods are attained when building 150 GW of each.

### Capacity distributions at minimal onshore wind and transmission grid

The aforementioned contour plots Figure 6, Figure 7 and 7 outline what is likely possible within specified cost ranges and subject to technology cost uncertainty but do not expose the changes the overall system layout experiences when reaching for the extremes in one technology. Therefore, we show in Figures 7D and 5E how the system-wide capacity distributions vary compared to the least-cost solutions (Figure 3) for two illustrative alternative objectives. For that, we choose the scenarios with least onshore wind capacity and least transmission expansion because they are often linked to the social acceptance of energy infrastructures.

Figure 7D illustrates that reducing onshore wind capacity is predominantly compensated for by increased offshore wind generation but also added solar capacities. The increased focus on offshore wind also leads to a tendency toward more hydrogen storage, while transmission expansion levels are similarly distributed as for the least-cost solutions. From Figure 7E we can further extract that avoiding transmission expansion entails more hydrogen storage that compensates balancing in space with balancing in time, and more generation capacity overall, where resources are distributed to locations with high demand but weaker capacity factors and more heavily curtailed.

## Discussion

In this work, we systematically explore a space of alternatives beyond least-cost solutions for society and politics to work with subject to uncertain technology cost projections. We show how narrowly following cost-optimal results underplays an immense degree of freedom in designing future renewable power systems. To make our finding that there is no unique path to cost-efficiency more robust, we account for technology cost uncertainties as one example of the many unknowns faced in the energy transition and draw the following conclusions.

### Wide range of trade-offs

We find that there is a substantial range of options within 8% of the least-cost fully renewable electricity system regardless of how cost developments will unfold. This holds across all technologies individually and even when considering dependencies between wind and solar, offshore and onshore wind, as well as hydrogen and battery storage as examples of flexibility options.

### Solutions to avoid

We also carve out parts of the solution space which are unlikely to keep costs within given cost ranges given the considered range of technology cost futures. For a fully renewable electricity system, either offshore or onshore wind capacities of the order of 600 GW along with some long-term storage technology and transmission network reinforcement appear essential in the scenarios we analyze. Less wind capacity leads to high-cost solutions in our model.

### Key technology cost sensitivities

We identify onshore wind cost as the apparent main determinant of system cost, though it can often be substituted with offshore wind for a small additional cost. This aligns with the finding that the near-optimal feasible space is flat. Moreover, the deployment of batteries is the most sensitive to its cost, whereas required levels of transmission expansion are least affected since transmission cost was not considered to be uncertain.

### Benefits of combining MGA and global sensitivity analysis

The combination of MGAs to explore the near-optimal solution space and global sensitivity analysis to account for an uncertain input parameter space unifies two approaches to uncertainty quantification. The presented methodology is helpful to show that near-optimal insights are robust to some uncertainty (in our case technology cost). Likewise, it can show whether some parts of the near-optimal solution space are more or less affected by uncertainty.

The robust finding of our study is that there is consistent investment flexibility in shaping fully renewable power systems, even without availing of the myriad flexibility options offered through sector coupling. This opens the floor to discussions about social trade-offs and navigating around issues, such as public opposition toward wind turbines or transmission lines. Rather than modelers making normative choices about how the energy system should be optimized, by applying multi-fidelity surrogate modeling techniques and the MGA methodology, we offer a methodology to present a wide spectrum of options and trade-offs that are feasible and within a reasonable cost range, to help society decide how to shape the future of the energy system.

### Limitations of the study

The need to solve models for many cost projections and near-optimal search directions in reasonable time means that compromises had to be made in other modeling dimensions. For instance, the analysis would profit from a richer set of technologies and further uncertain input parameters, including efficiencies of fuel cells and electrolysis or the consideration of concentrating solar power, geothermal energy, biomass, and nuclear to name just a few. All of these may also influence the near-optimal range of options for the technologies we considered. But as the number of considered technologies and parameters rises, so does the computational burden. Given the already considerable computational efforts involved in procuring our results, considering the full breadth of technologies and uncertainties would not have been feasible with the computational resources available. Moreover, further limitations apply to the scope of the analysis, which is limited to the electricity sector and does not consider rising electricity demand as also other energy sectors are electrified. Like a broader set of technologies, leveraging additional measures to integrate renewables through tighter cross-sectoral coupling and demand-side flexibilities would also yield different results about the technology choices for near-optimal energy system designs. Therefore, accounting for interactions across sectors at high resolution in a similar future study is desirable. Additionally, we assess no path dependencies via multi-period investments and endogenous learning but optimize for an emission reduction in a particular target system based on annualized costs. For computational reasons, we disregard interannual variations of weather data by basing the analysis just on a single weather year for computational reasons, as well as uncertainties about future demand predictions and electrification rates. Finally, aspects such as reserves, system adequacy, and inertia have not been considered.

## STAR★Methods

### Key resources table

**Table:** 

REAGENT or RESOURCE	SOURCE	IDENTIFIER
**Deposited data**		

PyPSA-Eur	Hörsch et al.^24^	https://www.github.com/pypsa/pypsa-eur

**Software and algorithms**		

PyPSA	Brown et al.^27^	https://github.com/pypsa/pypsa

### Resource availability

#### Lead contact

Requests for further information, resources and materials should be directed to the lead contact, Fabian Neumann (f.neumann@tu-berlin.de).

#### Materials availability

Not applicable.

### Method details

For the description of our experimental procedures, we first outline how we obtain least-cost and near-optimal solutions for a given cost parameter set. We then describe the model of the European power system and define the cost uncertainties. Finally, we explain how we make use of multi-fidelity surrogate modeling techniques based on polynomial chaos expansions and find an experimental design that efficiently covers the parameter space.

#### Least-cost investment planning

The objective of long-term power system planning is to minimize the total annual system costs, comprising annualized capital costs $c_{⋆}$ for investments at locations *i* in generator capacity $G_{i,r}$ of technology *r*, storage capacity $H_{i,s}$ of technology *s*, and transmission line capacities $F_{l}$, as well as the variable operating costs $o_{⋆}$ for generator dispatch $g_{i,r,t}$:

(Equation 1) $$\min_{G,H,F,g}\;\left\{\sum_{i,r}c_{i,r}\cdot G_{i,r}+\sum_{i,s}c_{i,s}\cdot H_{i,s}+\sum_{l}c_{l}\cdot F_{l}+\sum_{i,r,t}w_{t}\cdot o_{i,r}\cdot g_{i,r,t}\right\}$$

where the snapshots *t* are weighted by $w_{t}$ such that their total duration adds up to one year. The objective is subject to a set of linear constraints that define limits on (i) the capacities of infrastructure from geographical and technical potentials, (ii) the availability of variable renewable energy sources for each location and point in time, and (iii) linearized multi-period optimal power flow (LOPF) constraints including storage consistency equations, which we describe in more detail in the following.

The capacities of generation, storage and transmission infrastructure are limited to their geographical potentials from above and existing infrastructure from below:

(Equation 2) $${\underline{G}}_{i,r}\leq G_{i,r}\leq {\bar{G}}_{i,r}\;\forall i,r$$

(Equation 3) $${\underline{H}}_{i,s}\leq H_{i,s}\leq {\bar{H}}_{i,s}\;\forall i,s$$

(Equation 4) $${\underline{F}}_{l}\leq F_{l}\leq {\bar{F}}_{l}\forall l$$

The dispatch of a renewable generator is constrained by its rated capacity and the time- and location-dependent availability ${\bar{g}}_{i,r,t}$, given in per-unit of the generator’s capacity:

(Equation 5) $$0\leq g_{i,r,t}\leq {\bar{g}}_{i,r,t}G_{i,r}\;\forall i,r,t$$

The dispatch of storage units is described by a charge variable $h_{i,s,t}^{+}$ and a discharge variable $h_{i,s,t}^{-}$, each limited by the power rating $H_{i,s}$.

(Equation 6) $$0\leq h_{i,s,t}^{+}\leq H_{i,s}\;\forall i,s,t$$

(Equation 7) $$0\leq h_{i,s,t}^{-}\leq H_{i,s}\;\forall i,s,t$$

The energy levels $e_{i,s,t}$ of all storage units are linked to the dispatch by

(Equation 8) $$e_{i,s,t}=\eta_{i,s,0}^{w_{t}}\cdot e_{i,s,t-1}+w_{t}\cdot h_{i,s,t}^{\text{inflow}}-w_{t}\cdot h_{i,s,t}^{\text{spillage}}\forall i,s,t+\eta_{i,s,+}\cdot w_{t}\cdot h_{i,s,t}^{+}-\eta_{i,s,-}^{-1}\cdot w_{t}\cdot h_{i,s,t}^{-}\text{.}$$

Storage units can have a standing loss $\eta_{i,s,0}$, a charging efficiency $\eta_{i,s,+}$, a discharging efficiency $\eta_{i,s,-}$, natural inflow $h_{i,s,t}^{\text{inflow}}$ and spillage $h_{i,s,t}^{\text{spillage}}$. The storage energy levels are assumed to be cyclic and are constrained by their energy capacity

(Equation 9) $$e_{i,s,0}=e_{i,s,T}\;\forall i,s$$

(Equation 10) $$0\leq e_{i,s,t}\leq {\bar{T}}_{s}\cdot H_{i,s}\;\forall i,s,t\text{.}$$

To reduce the number of decisison variables, we link the energy capacity to power ratings with a technology-specific parameter ${\bar{T}}_{s}$ that describes the maximum duration a storage unit can discharge at full power rating. Owing to the absence of large thermal power plants in our fully renewable scenarios, unit commitment constraints regarding the ramping, start-up and shut-down of generators are not considered.

Kirchhoff’s Current Law (KCL) requires local generators and storage units as well as incoming or outgoing flows $f_{l,t}$ of incident transmission lines l to balance the inelastic electricity demand $d_{i,t}$ at each location *i* and snapshot *t*

(Equation 11) $$\sum_{r}g_{i,r,t}+\sum_{s}h_{i,s,t}+\sum_{l}K_{il}f_{l,t}=d_{i,t}\forall i,t\text{,}$$

where $K_{il}$ is the incidence matrix of the network.

Kichhoff’s Voltage Law (KVL) imposes further constraints on the flow of AC lines. Using linearized load flow assumptions, the voltage angle difference around every closed cycle in the network must add up to zero. We formulate this constraint using a cycle basis $C_{lc}$ of the network graph where the independent cycles *c* are expressed as directed linear combinations of lines l.^28^ This leads to the constraints

(Equation 12) $$\sum_{l}C_{lc}\cdot x_{l}\cdot f_{l,t}=0\forall c,t$$

where $x_{l}$ is the series inductive reactance of line l. Controllable HVDC links are not affected by this constraint.

Finally, all line flows $f_{l,t}$ must be operated within their nominal capacities $F_{l}$

(Equation 13) $$\left|f_{l,t}\right|\leq {\bar{f}}_{l}F_{l}\;\forall l,t\text{,}$$

where ${\bar{f}}_{l}$ acts as a per-unit buffer capacity to protect against the outage of single circuits.

This problem is implemented in the open-source tool PyPSA^27^ and is solved by Gurobi. Note, that it assumes perfect foresight for a single reference year based on which capacities are optimized. It does not include pathway optimization, nor aspects of reserve power, or system stability. Changes of line expansion to line impedance are ignored.

#### Finding near-optimal alternatives

Using the least-cost solution as an anchor, we use the ϵ-constraint method from multi-objective optimization to find near-optimal feasible solutions.^6^^,^^29^ For notational brevity, let $c^{⊤}x$ denote the linear objective function Equation 1 and Ax≤b the set of linear constraints (Equation 2), (Equation 3), (Equation 4), (Equation 5), (Equation 6), (Equation 7), (Equation 8), (Equation 9), (Equation 10), (Equation 11), (Equation 12), (Equation 13) in a space of continuous variables, such that the minimized system cost can be represented by

(Equation 14) $$C=\min_{x}\left\{c^{⊤}x|Ax\leq b\right\}\text{.}$$

We then encode the original objective as a constraint such that the cost increase is limited to a given ϵ. In other words, the feasible space is cut to solutions that are at most ϵ more expensive than the least-cost solution. Given this slack, we can formulate alternative search directions in the objective. For instance, we can seek to minimize or maximize the sum of solar installations $x_{s}\subseteq x$ with

(Equation 15) $$\underline{x_{s}}=\min_{x_{s}}\left\{1^{⊤}x_{s}|Ax\leq b,c^{⊤}x\leq \left(1+\epsilon \right)\cdot C\right\}$$

(Equation 16) $$\bar{x_{s}}=\max_{x_{s}}\left\{1^{⊤}x_{s}|Ax\leq b,c^{⊤}x\leq \left(1+\epsilon \right)\cdot C\right\}\text{.}$$

To draw a full picture of the boundaries of the near-optimal feasible space, we systematically explore the extremes of various technologies: we both minimize and maximize the system-wide investments in solar, onshore wind, offshore wind, any wind, hydrogen storage, and battery storage capacities, as well as the total volume of transmission network expansion. Evaluating each of these technology groups for different cost deviations ϵ∈{1%,2%,4%,6%,8%} allows us to observe how the degree of freedom regarding investment decisions rises as the optimality tolerance is increased, both at lower and upper ends. The boundaries delineate Pareto frontiers on which no criterion, neither reducing system cost nor extremizing the capacity of a technology, can be improved without depressing the other. By arguments of convexity, these extremes even define limits within which all near-optimal solutions are contained. Moreover, although this scheme primarily studies aggregated capacities, the solutions are spatially explicit, and we can inspect for each case how the capacities of each technology are distributed within the network.

The near-optimal analysis above only explores the extremes of one technology at a time, i.e. one direction in the feasible space. But actually the space of attainable solutions within ϵ of the cost-optimum is multi-dimensional. To further investigate trade-offs between multiple technologies, in addition to the ϵ-constraint and the objective to extremize capacities of a particular technology, we formulate a constraint that fixes the capacity of another technology within its bounds for a given ϵ. For instance, we search for the minimum amount of wind capacity $x_{w}\subseteq x$ given that a certain amount of solar is built

(Equation 17) $$\underline{x_{w}}=\min_{x_{w}}\left\{1^{⊤}x_{w}|Ax\leq b,c^{⊤}x\leq \left(1+\epsilon \right)\cdot C,1^{⊤}x_{s}=\underline{x_{s}}+\alpha \cdot \left(\bar{x_{s}}-\underline{x_{s}}\right)\right\}\text{,}$$

and correspondingly for the maximum $\bar{x_{w}}$. The α denotes the relative position within the near-optimal range of the second technology at given ϵ, in this case the solar capacities. For example, at α=0% we look for the least wind capacity given that minimal solar capacities are built for the given ϵ. An alternative but more complex approach to spanning the space of near-optimal solutions in multiple dimensions at a time using a quick hull algorithm was presented by Pedersen et al.^16^

Due to computational constraints, we focus on technologies which are assumed to lend themselves to substitution and limit the corresponding analysis to a single illustrative cost increase level of ϵ=6%. The same methodology can be applied to any other value for ϵ. We consider the three pairs, (i) wind and solar, (ii) offshore and onshore wind, (iii) hydrogen and battery storage, by minimizing and maximizing the former while fixing the latter at positions α∈{0%,25%,50%,75%,100%} within the respective near-optimal range.

#### Open electricity sector model PyPSA-Eur

The instances of the coordinated capacity expansion problem (see least-cost investment planning) are based on PyPSA-Eur, which is an open model of the European power transmission system that combines high spatial and temporal resolution.^24^ Because it only uses open data and every processing step is defined in a workflow,^30^ we achieve a high level of transparency and reproducibility. In the following, we outline the main features and configurations, and refer to the supplementary material and Hörsch et al.^24^ for more details.

#### Scenario

We target a fully renewable electricity system based on variable resources such as solar photovoltaics, onshore wind and offshore wind, that has not carbon emissions. We pursue a greenfield approach subject to a few notable exceptions. The existing hydro-electric infrastructure (run-of-river, hydro dams, pumped-storage) is included but not considered to be extendable due to assumed geographical constraints. Furthermore, the existing transmission infrastructure can only be reinforced continuously but may not be removed. In addition to balancing renewables in space with transmission networks, the model includes storage options at each node to balance renewables in time. We consider two extendable storage technologies: battery storage representing short-term storage suited to balancing daily fluctuations and hydrogen storage which exemplifies long-term synoptic and seasonal storage. We do not consider any further sector-coupling technologies or demand-side flexibilities, but expect that these could substitute for some of the storage requirements.

#### Spatial and temporal resolution

Since the spatial and temporal resolution strongly affects the size of the optimization problem, running the model at full resolution is computationally infeasible. In our analysis, we therefore make use of two levels of aggregation, reflecting a compromise between the computational burden incurred by high-resolution models and the growing inaccuracies regarding transmission bottlenecks and resource distribution in low-resolution models. We consider a low-fidelity model with 37 nodes at a 4-hourly resolution for a full year that models power flow via a transport model (i.e. excluding KVL of Equation 12) and a high-fidelity model with 128 nodes at a 2-hourly resolution that is subject to linearized load flow constraints (Figure 1). More information on how the results of two models with varying resolution are merged is provided in the section multi-fidelity approach in surrogate modeling.

#### Transmission grid and hydro-electricity

The topology of the European transmission network is retrieved from the ENTSO-E transparency map and includes all lines at and above 220 kV. Capacities and electrical characteristics of transmission lines and substations are inferred from standard types for each voltage level, before they are transformed to a uniform voltage level. For each line, N−1 security is approximated by limiting the line loading to 70% of its nominal rating. The dataset further includes existing high-voltage direct current (HVDC) links and planned projects from the Ten Year Network Development Plan (TYNDP). Existing run-of-river, hydro-electric dams, pumped-hydro storage plants are retrieved from *powerplantmatching*, a merged dataset of conventional power plants.

#### Renewable energy potentials

Eligible areas for developing renewable infrastructure are calculated per technology and the grid nodes’ Voronoi cells, assuming wind and solar installations always connect to the closest substation. How much wind and solar capacity may be built at a location is constrained by eligible codes of the CORINE land use database and is further restricted by distance criteria, allowed deployment density, and the natural protection areas specified in the NATURA 2000 dataset. Moreover, offshore wind farms may not be developed at sea depths exceeding 50 m, as indicated by the GEBCO bathymetry dataset.

#### Renewables and demand time series

The location-dependent renewables availability time series are generated based on two historical weather datasets for the year 2013, which is an average year in terms of wind and solar availability. We retrieve wind speeds, run-off and surface roughness from the ERA5 reanalysis dataset and use the satellite-aided SARAH-2 dataset for the direct and diffuse surface solar irradiance. Models for wind turbines, solar panels, and the inflow into the basins of hydro-electric dams convert the weather data to hourly capacity factors and aggregate these to each grid node. Historical country-level load time series are taken from ENTSO-E statistics and are heuristically distributed to each grid node to 40% by population density and to 60% by gross domestic product.

#### Technology cost uncertainty

Uncertainty of technology cost projections is driven by two main factors: unknown learning rates (i.e. how quickly costs fall as more capacity is built) and unclear deployment rates (i.e. how much capacity will be built in the future).^31^^,^^32^ As modeling technological learning endogeneously is computationally challenging due to the nonconvexity it entails,^33^^,^^34^^,^^35^ technology cost uncertainty is typically defined exogenously by an interval within which costs may vary and a distribution that specifies which segments are more probable.

Ranges of cost projections are best chosen as wide as possible to avoid excluding any plausible scenarios.^9^^,^^36^ When uncertainty has been considered in the literature, cost assumptions have commonly been modeled to vary between ±20% and ±65% depending on the technology’s maturity.^3^^,^^21^^,^^36^^,^^37^^,^^38^ In this study, we consider uncertainty regarding the annuities of onshore wind, offshore wind, solar PV, battery and hydrogen storage systems. The latter comprises the cost of electrolysis, cavern storage, and fuel cells. For solar PV we assume an even split between utility-scale PV and residential rooftop PV. Evaluating uncertainties based on annuities has a distinct advantage. They can be seen to simultaneously incorporate uncertainties about the overnight investments, fixed operation and maintenance costs, their lifetime, and the discount rate, since multiple combinations lead to the same annuity. We built the uncertainty ranges presented in Table 1 from the optimistic and pessimistic technology cost and lifetime projections for the year 2050 from the Danish Energy Agency, which correspond to 90% confidence intervals.^25^ In cases where no uncertainty ranges were provided for the year 2050, such as for rooftop PV, projections for the year 2030 define the upper end of the uncertainty interval.

Distributions of cost projections have been assumed to follow normal^20^ or triangular^38^ distributions. But independent uniform distributions are the most prevalent assumption.^3^^,^^12^^,^^36^^,^^37^^,^^39^^,^^40^^,^^41^^,^^42^ This approach is backed by the maximum entropy approach,^3^ which states that given the persistent lack of knowledge about the distribution the independent uniform distribution, that makes fewest assumptions, is most appropriate. Although the assumed independence may neglect synergies between technologies, for example, between offshore and onshore wind turbine development, we follow the literature by assuming that the cost are independent and uniformly distributed within the ranges specified in Table 1.

#### Surrogate modeling with polynomial chaos expansion

Searching for least-cost solutions (see least-cost investment planning) and many associated near-optimal alternatives (see finding near-optimal alternatives) of a highly resolved power system model (see open electricity sector model PyPSA-Eur) on its own is already labour-intensive from a computational perspective. Repeating this search for a large variety of cost assumptions (see technology cost uncertainty), to be able to make statements about the robustness of investment flexibility near the optimum under uncertainty, adds another layer to the computational burden.

Surrogate models offer a solution for such cases, where the outcome of the original model cannot be obtained easily. Surrogate names are also known by names such as approximation models, response surface methods, metamodels and emulators. In contrast to the full model, they only imitate the input/output behavior for a selection of aggregated outputs, but take much less time to compute.^43^ Like other machine learning techniques, they generalize from a training dataset that comprises only a limited number of samples. As surrogate models interpolate gaps in the parameter space that are not contained in the sample set, which would otherwise be computationally expensive to fill, they are well suited to use cases such as parameter space exploration and sensitivity analysis.

Consequently, in this paper we make use of surrogate models that map the cost of onshore wind, offshore wind, solar, hydrogen, and battery storage (Table 1) onto a selection of eight system-level outputs. These are the total system cost and the installed onshore wind, offshore wind, solar, hydrogen, battery, and transmission network capacities. We construct surrogate models for least-cost and near-optimal solutions separately for each system cost slack, search direction, fixed total capacity, and output variable. This results in a collection of 808 individual surrogate models based on 101 solved optimization problems per set of cost assumptions. The method we choose from an abundance of alternatives is based on polynomial chaos expansion (PCE).^26^^,^^44^^,^^45^ We select this approach because the resulting approximations allow efficient analytical statistical evaluation^26^ and can conveniently combine training data from variously detailed models.^43^

The general idea of surrogate models based on PCE is to represent uncertain model outputs as a linear combination of orthogonal basis functions of the random input variables weighted by deterministic coefficients.^46^ It is a Hilbert space technique that works in principle analogously to decomposing a periodic signal into its Fourier components.^46^ Building the surrogate model consists of the following steps: (i) sampling a set of cost projections from the parameter space, (ii) solving the least-cost or near-optimal investment planning problem for each sample, (iii) selecting an expansion of orthogonal polynomials within the parameter space, (iv) performing a regression to calculate the polynomial coefficients, and ultimately (v) using the model approximation for statistical analysis. In the following, we formalize this approach mathematically, which we implemented using the *chaospy* toolbox,^47^ and elaborate on individual aspects in more detail.

We start by defining the vector of random input variables as

(Equation 18) $$x=\left\{x_{1},\ldots ,x_{m}\right\}$$

that represents the *m* uncertain cost projections. Further, we let

(Equation 19) y=f(x)

describe how the uncertainty of inputs x propagates through the computationally intensive model *f* (i.e. the solving of a large capacity expansion problem) to the outputs y∈R.

We can represent the computational model *f* with its polynomial chaos expansion

(Equation 20) $$y=f\left(x\right)=\sum_{\alpha \in N^{m}}r_{\alpha }\psi_{\alpha }\left(x\right)\text{,}$$

where $\psi_{\alpha }$ denotes multivariate orthogonal polynomials that form a Hilbertian basis and $r_{\alpha }\in R$ are the corresponding polynomial coefficients.^26^ The multiindex $\alpha =\left\{\alpha_{1},\ldots ,\alpha_{m}\right\}$ denotes the degree of the polynomial $\psi_{\alpha }$ in each of the *m* random input variables $x_{i}$. As Equation 20 features an infinite number of unknown coefficients, it is common practice to approximate by truncating the expansion to get a finite number of coefficients

(Equation 21) $$f\left(x\right)\approx f^{\prime }\left(x\right)=\sum_{\alpha \in A^{m,p}}r_{\alpha }\psi_{\alpha }\left(x\right)\text{.}$$

In the standard truncation scheme,^26^^,^^45^ all polynomials in *m* input variables (i.e. cost uncertainties) where the total degree is less than a user-defined *p* are selected. We can write this as a set of indices

(Equation 22) $$A^{m,p}=\left\{\alpha \in N^{m}:\left|\alpha \right|\leq p\right\}\text{,}$$

where $\left|\alpha \right|=\sum_{i=1}^{m}\alpha_{i}$. Given the joint distribution of cost uncertainties of x and a maximum degree, a suitable collection of orthogonal polynomials can be constructed using a three terms recurrence algorithm.^47^ The cardinality of the truncated PCE,

(Equation 23) $$q=\text{card}\;A^{m,p}=\left(\begin{array}{l} m+p\\ p \end{array}\right)=\frac{\left(m+p\right)!}{m!p!}\text{,}$$

indicates the number of unknown polynomial coefficients.

We determine these coefficients by a regression based on a set of cost parameter samples and the corresponding outputs,

(Equation 24) $$X=\left\{x^{\left(1\right)},\ldots ,x^{\left(n\right)}\right\}\;\text{and}\;Y=\left\{f\left(x^{\left(1\right)}\right),\ldots ,f\left(x^{\left(n\right)}\right)\right\}\text{.}$$

Using this training dataset, we minimize the least-square residual of the polynomial approximation across all observations. We add an extra $L_{1}$ regularization term, that induces a preference for fewer non-zero coefficients, and solve

(Equation 25) $$\hat{r}=\underset{r\in R^{q}}{\mathrm{argmin}}\left[\frac{1}{n}\sum_{i=1}^{n}{\left(f\left(x^{\left(i\right)}\right)-\sum_{\alpha \in A^{m,p}}r_{\alpha }\psi_{\alpha }\left(x^{\left(i\right)}\right)\right)}^{2}+\lambda ∥r{∥}_{1}\right]\text{,}$$

where we set the regularization penalty to λ=0.005. This results in a sparse PCE that has proven to improve approximations in high-dimensional uncertainty spaces and to reduce the required number of samples for comparable approximation errors.^45^ Knowing the optimized regression coefficients, we can now assemble the complete surrogate model

(Equation 26) $$y=f\left(x\right)\approx f^{\prime }\left(x\right)=\sum_{\alpha \in A^{m,p}}{\hat{r}}_{\alpha }\psi_{\alpha }\left(x\right)\text{.}$$

#### Multi-fidelity approach in surrogate modeling

To construct a sufficiently precise PCE-based surrogate model, it is desirable to base it on many samples from a high-fidelity model. However, this is likely prohibitively time-consuming. On the other hand, relying only on samples from a low-fidelity model may be too inaccurate.^48^ For example, an investment model that features only a single node per country will underestimate transmission bottlenecks and regionally uneven resource or demand distribution. In the section on Open Electricity Sector Model PyPSA-Eur we already alluded to using two models with varying spatial and temporal resolution in this paper. We integrate both in a multi-fidelity approach,^43^^,^^48^ and demonstrate how we can simultaneously avail of high coverage of the parameter space by sampling the simpler model many times, and the high spatiotemporal detail yielded by fewer more complex model runs.

The idea of the multi-fidelity approach is to build a corrective surrogate model $f_{\Delta }^{\prime }\left(x\right)$ for the error of the low-fidelity model $f_{l}$ compared to the high-fidelity model $f_{h}$

(Equation 27) $$f_{\Delta }\left(x\right)=f_{h}\left(x\right)-f_{l}\left(x\right)\text{,}$$

and add it to a surrogate model of the low-fidelity model to approximate the behavior of the high-fidelity model

(Equation 28) $$f_{h}^{\prime }\left(x\right)=f_{l}^{\prime }\left(x\right)+f_{\Delta }^{\prime }\left(x\right)\text{.}$$

Typically, the corrective PCE rectifies only the lower order effects of the low-fidelity surrogate model (e.g. linear effects of an individual technology’s cost).^43^ The advantage is that this way the correction function can be determined based on fewer samples analogous to the previous section surrogate modeling with polynomial chaos expansion. To sample the errors, it is only required that the high-fidelity samples are a subset of the low-fidelity samples, e.g.

(Equation 29) $$X_{h}=\left\{x^{\left(1\right)},\ldots ,x^{\left(n_{h}\right)}\right\}\;\text{and}\;X_{l}=\left\{x^{\left(1\right)},\ldots ,x^{\left(n_{h}\right)},\ldots ,x^{\left(n_{l}\right)}\right\}\text{,}$$

which we can easily guarantee by using deterministic low-discrepancy series in the experimental design (see experimental design for surrogate modeling). With $p_{\Delta }<p_{l}$ and consequently $A_{\Delta }\subset A_{l}$, the multi-fidelity surrogate model can be written as a combination of low-fidelity and corrective polynomial coefficients

(Equation 30) $$f_{h}^{\prime }\left(x\right)=\sum_{\alpha \in A_{l}^{m,p_{l}}\cap A_{\Delta }^{m,p_{\Delta }}}\left(r_{l,\alpha }+r_{\Delta ,\alpha }\right)\psi_{\alpha }\left(x\right)+\sum_{\alpha \in A_{\ell }^{m,p_{\ell }}\setminus A_{\Delta }^{m,p_{\Delta }}}r_{l,\alpha }\psi_{\alpha }\left(x\right)\text{.}$$

In this work, we apply a multi-fidelity surrogate model that considers effects up to order three observed in the low-fidelity model. These are then corrected with linear terms derived from insights from the high-fidelity model. We justify this choice by experimentation in the Validation of the Surrogate Models, by testing against other typical choices between orders one to five.^45^ Given the polynomial expansion order, the remaining question is how many samples are necessary to attain an acceptable approximation.

#### Experimental design for surrogate modeling

The experimental design covers strategies to find sufficiently high coverage of the parameter space at low computational cost.^23^^,^^44^ It deals with how many samples are drawn and what sampling method is used.

Traditional Monte-Carlo sampling with pseudo-random numbers is known to possess slow convergence properties, especially in high-dimensional parameter spaces. So-called low-discrepancy series can greatly improve on random sampling. Because they are designed to avoid forming large gaps and clusters, these deterministic sequences efficiently sample from the parameter space.^44^ Thus, we choose to draw our samples from a low-discrepancy Halton sequence.

For the question about how many samples should be drawn, we resort to the oversampling ratio (OSR) as a guideline. The OSR is defined as the ratio between the number of samples and the number of unknown coefficients.^43^ The literature recommends values between two and three.^43^^,^^44^^,^^45^^,^^49^ In other words, for a sufficiently accurate approximation, there should be significantly more samples than unknown coefficients. If the OSR is lower, the regression is prone to the risk of overfitting. On the other hand, a high OSR may lead to a very coarse approximation.^43^

According to Equation 23, targeting an OSR of two and considering the five uncertain technology cost parameters (Table 1), approximating linear effects would require at least 12 samples, whereas cubic relations would already need 112 samples. Even 504 samples would be necessary to model the dynamics of order 5. To investigate the quality of different PCE orders and retain a validation dataset, we draw 500 samples for the low-fidelity model. Due to the computational burden carried by the high-fidelity models, we settle on a linear correction in advance, such that 15 samples for the high-fidelity model are acceptable. In combination with 101 least-cost and near-optimal optimization runs for each sample, this setup results in a total number of 50,500 runs of the low-fidelity model and 1,515 runs of the high-fidelity model. On average a single high-fidelity model run took 20 GB of memory and 5 h to solve. Each low-fidelity model run on average consumed 3 GB of memory and completed within 5 min. This setup profits tremendously from parallelization as it involves numerous independent optimization runs. Moreover, it would have been infeasible to carry out without high-performance computing.

#### Validation of the surrogate models

We justify the use of surrogate modeling by cross-validation. Out of the 500 low-fidelity samples, 100 samples are not used in the regression. This validation dataset is unknown to the surrogate model and is consulted to assess the approximation’s quality. Because the high-fidelity sample size is limited and approximating near-optimal solutions is not assumed to fundamentally differ, we base the validation on low-fidelity least-cost solutions only. We experimentally evaluate the approximation errors between predicted and observed data for different combinations of polynomial order and sample size to decide on a suitable parameterization. We present the coefficient of determination ($R^{2}$) for the variance captured, the mean absolute (percentage) errors (MAE/MAPE) for absolute and relative deviations, and the root mean squared error (RMSE).

Regarding the number of samples required, Figure 2 foremost illustrates that, given enough samples, we achieve average relative errors of less than 4% for most output variables. This is comparable to the cross-validation errors from Tröndle et al.^3^ at rates below 5%. Only for offshore wind and battery storage, we observe larger errors. However, this can be explained by a distortion of the relative measure when these technologies are hardly built for some cost projections. On the contrary, the prediction of total system costs is remarkably accurate. Figure 2 also demonstrates that for a polynomial order of 3, we gain no significant improvement with more than 200 samples. In fact, thanks to the regularization term used in the regression, we already attain acceptable levels of accuracy with as few as 50 samples. Moreover, the high $R^{2}$ values underline that the surrogate model can explain most of the output variance.

Regarding the polynomial order, Figure 2 shows that an order of 2 and below may be too simple to capture the interaction between different parameters. On the other hand, an order of 4 and above yields no improvement and, were it not for the moderating regularization term, would even result in a loss of generalization properties due to overfitting. As higher-order approximations require significantly more samples, an order of 3 appears to be a suitable compromise to limit the computational burden.

## Data Availability

The code to reproduce the experiments as well as results dat including selected networks and all graphics is available at github.com/fneum/broad-ranges and archived at https://doi.org/10.5281/zenodo.6641551. We also refer to the documentation of PyPSA (pypsa.readthedocs.io) and PyPSA-Eur (pypsa-eur.readthedocs.io).
